# Factors influencing patient-reported outcomes in operatively managed tibial plateau fractures

**DOI:** 10.1007/s00590-026-04687-0

**Published:** 2026-02-26

**Authors:** Doriann Alcaide, Robin Litten, Jeffrey Clay Krout, Garrett Hawkins, Alexa Smitherman, Ryan McIlwain, Gerald McGwin, Clay Spitler, Joey Johnson

**Affiliations:** 1 Department of Orthopaedic Surgery, University of Missouri–Kansas City, Kansas City, USA; 2 Department of Orthopaedic Surgery, University of Alabama at Birmingham, Birmingham, USA; 3 Heersink School of Medicine, University of Alabama at Birmingham, Birmingham, USA

**Keywords:** Tibial plateau fracture, Patient-reported outcomes, High-energy trauma, Injury severity score

## Abstract

**Purpose:**

Tibial plateau fractures are complex injuries with high complication rates, yet their broader impact on patient-reported function and quality of life remains underexplored. This study assessed the impact of demographics, fracture patterns, and treatment characteristics on patient-reported outcomes measurement information system (PROMIS) scores in patients who underwent surgical fixation of tibial plateau fractures.

**Methods:**

A retrospective cohort study was conducted at a Level I trauma center (2022–2024) including adults with operatively treated tibial plateau fractures who completed 6-month PROMIS surveys. Exclusions were age < 18 years, non-operative or percutaneous fixation, and inadequate medical record documentation. Primary outcomes were 6-month scores for PROMIS physical function (PF), pain interference (PI), global physical health (GPH), global mental health (GMH), anxiety/depression, brief resilience scale (BRS), and percent of normal function. Outcomes were compared by sex, age, mechanism of injury, fracture pattern, Schatzker type, and external fixation.

**Results:**

Of 413 patients, 106 met inclusion criteria. Mean age was 50.4 years and 51.9% were male. Patients < 50 reported higher PI (62.7 vs. 59.3, *p* = 0.017) and lower GPH (38.8 vs. 42.4, *p* = 0.023). Among females, those < 50 had higher PI (64.0 vs. 58.5, *p* = 0.009), lower GPH (37.5 vs. 43.1, *p* = 0.011), and lower anxiety (60.2 vs. 64.1, *p* = 0.046). After adjustment, high-energy injuries were associated with worse GPH (*p* = 0.014), GMH (*p* = 0.011), depression (*p* = 0.009), anxiety (*p* = 0.015), percent of normal (*p* = 0.002), and BRS (*p* = 0.004).

**Conclusion:**

Early recovery after surgically treated tibial plateau fractures varies by patient and injury characteristics: younger patients, particularly females under 50, reported worse pain and global physical health, while high-energy mechanisms were independently associated with poorer physical and psychological outcomes across multiple domains.

**Supplementary Information:**

The online version contains supplementary material available at 10.1007/s00590-026-04687-0.

## Introduction

Fractures of the tibial plateau can be devastating injuries that account for up to 8% of fractures in the elderly and 1% of all fractures [1–4]. They are associated with relatively high complication rates including 2–20% for nonunion, 3–26% for infection, and 21–44% for post-traumatic osteoarthritis [5–7]. Although previous literature has highlighted adverse clinical outcomes, the extent to which these injuries impact patient function and quality of life remains less well understood.

The patient-reported outcomes measurement information system (PROMIS), developed by the National Institutes of Health, is designed to assess a patient’s perception of their health and quality of life [8–10]. It has been adopted in clinical practice to track outcomes after interventions across domains such as pain interference, physical function, global health, depression, and anxiety [9, 10]. As its use expands in orthopaedics, PROMIS offers a standardized framework to compare injury patterns, operative approaches, and patient characteristics in relation to functional outcomes, helping to identify factors associated with recovery [11].

Although prior studies have used PROMIS scores to evaluate outcomes in tibial plateau fractures, most have focused on fixation techniques for bicondylar patterns or specific injury characteristics, including tubercle involvement or concurrent meniscal repair [12–16]. Moreover, these studies have largely focused on physical function and pain interference PROMIS domains [12, 14, 15].

With the growing use of PROMIS scores in orthopedic trauma, evaluating the influence of non-modifiable risk factors on both physical function and mental health has become increasingly important [17]. This study aimed to assess the impact of patient demographics, fracture patterns, and treatment characteristics on 6-month PROMIS scores in patients who underwent surgical fixation of tibial plateau fractures. It was hypothesized that younger age, female sex, or high-energy mechanisms of injury may be associated with lower PROMIS, percent of normal, or BRS scores.

## Methods

### Study design, setting, and participants

Following institutional review board approval, a retrospective review was conducted to identify patients with tibial plateau fractures (AO/OTA 41) at a Level I trauma center from July 2022 to May 2024. Patients were identified using Current Procedural Terminology codes 27535 and 27536.

Patients who sustained tibial plateau fractures and underwent open reduction internal fixation (ORIF) were included. Patients were excluded if they were under 18 years of age, had insufficient medical record documentation, were managed non-operatively or with closed reduction percutaneous fixation, or did not complete 6-month PROMIS surveys.

PROMIS is a standardized question bank developed by the National Institute of Health to measure patient-reported outcomes (PROs), including physical, mental, and social health across many medical conditions and disease states, including disease and pathology of the musculoskeletal system [10]. PROMIS scores are reported on a T-score metric standardized to the U.S. general population (mean = 50, standard deviation = 10). For physical function (PF), global physical health (GPH), and global mental health (GMH), higher scores indicate better health, whereas for pain interference (PI), anxiety, and depression, higher scores indicate worse symptoms.

The Brief Resilience Scale (BRS) is a validated six-item questionnaire scored on a 5-point Likert scale (1 = strongly disagree to 5 = strongly agree) designed to assess an individual’s ability to recover from stress, and percent of normal function is a single-item global assessment in which patients estimate their current level of function as a percentage of their pre-injury status (scaled 0–100%) [18, 19]. PROMIS, percent of normal function, and BRS surveys were administered electronically and sent directly to patients. All surveys utilized in this study are available in the Supplemental Digital Content.

The primary outcomes were 6-month patient-reported outcome scores for PF, PI, GMH, GPH, anxiety, depression, BRS and percent of normal function. Six months was selected as the primary survey timepoint because it yielded the highest response rate and offered a practical reflection of early functional recovery. Follow-up beyond 6 months was limited due to well-documented frequent loss to follow-up among orthopaedic trauma patients [20, 21].

Demographic variables including sex, age, race, and body mass index (BMI) were collected. Comorbidities were also noted, including diabetes mellitus (DM), hypertension (HTN), tobacco use, alcohol use, and American Society of Anesthesiologists (ASA) score. Injury-related variables included laterality, mechanism of injury, open versus closed injury, and Schatzker classification. Additional injury characteristics collected included polytrauma status, presence of an ipsilateral lower extremity injury, and use of an external fixation. Polytrauma was defined as an Injury Severity Score (ISS) greater than 15 [22, 23]. All variables were collected via retrospective review of the electronic medical record.

PROMIS, BRS, and percent of normal scores were compared by sex, age group, injury mechanism (high- vs. low-energy), fracture type (unicondylar vs. bicondylar), Schatzker classification, and use of external fixation. High-energy mechanisms of injury were defined as those including motor vehicle collisions (MVC), motorcycle collisions (MCC), falls from height (FFH), gunshot wounds (GSW), pedestrian-versus-automobile accidents (peds vs. auto), and crush injuries. Low-energy mechanisms were defined as falls from standing (FFS) and other miscellaneous, low-impact injuries.

### Statistical analyses

Descriptive statistics were generated to characterize the study population. Categorical variables were presented as numbers and percentages and were compared using chi-square or Fisher’s exact test. Continuous variables were reported with means and standard deviation and were compared using independent t-tests. To further evaluate the relationship between mechanism of injury and patient-reported outcomes, multivariable linear regression models were performed, adjusting for age, sex, and injury burden, measured by ISS. Two-sided *p* values of ≤ 0.05 were considered statistically significant. All statistical analyses were performed with IBM SPSS (Version 29.0.2.0).

## Results

Of 413 patients with tibial plateau fractures treated at this institution during the study period, 106 completed 6-month patient-reported outcome surveys and were included in the analysis. The mean age of this cohort was 50.4 years (range 19–81 years), and the majority (51.9%) were male. Most patients identified as white (70.8%), with a mean BMI of 30.7 kg/m^2^ (range 17.2–54.92 kg/m^2^). Hypertension was documented in 48 patients (45.3%), diabetes mellitus in 21 (19.8%), tobacco use in 31 (29.2%), and alcohol use in 57 (53.8%). Most patients were classified as ASA III (63.2%) (Table 1).

**Table 1:** Demographic and clinical characteristics among 106 patients with tibial plateau fractures

Variable	n (%) or mean (SD)
Age	50.4 (14.4)
Sex	
Male	55 (51.9)
Female	51 (48.1)
Follow up (days)	261.5 (136.0)
Race	
White	75 (70.8)
Black	26 (24.5)
Other	5 (4.7)
Body mass index (kg/m^2^)	30.8 (7.8)
Diabetes	21 (19.8)
Hypertension	48 (45.3)
Tobacco use	31 (29.2)
Alcohol use	57 (53.8)
ASA score	
1	3 (2.8)
2	31 (29.2)
3	67 (63.2)
4	5 (4.7)

*SD* standard deviation, *ASA* American Society of Anesthesiologists, *kg/m*^*2*^ kilograms per meters squared

The most common mechanism of injury in this cohort was motor vehicle collision (41.5%). Open fractures were present in 25.5% of patients. The majority sustained Schatzker type VI fractures (63.2%). Almost half (45.3%) were polytrauma patients, and 66.0% had associated ipsilateral lower extremity injuries. External fixation was used in 52.8% of cases (Table 2).

**Table 2:** Injury characteristics among 106 patients with tibial plateau fractures

Variable	Count (%)
Laterality	
Right	49 (46.2)
Left	57 (53.8)
Mechanism of injury	
MVC	44 (41.5)
MCC	12 (11.3)
FFS	14 (13.2)
FFH	17 (16.0)
GSW	3 (2.8)
Peds versus auto	8 (7.8)
Crush injury	5 (4.7)
Other	3 (2.8)
Open injury	27 (25.5)
Polytrauma	48 (45.3)
Schatzker classification	
I	5 (4.7)
II	24 (22.6)
III	5 (4.7)
IV	5 (4.7)
V	0 (0)
VI	67 (63.2)
Ipsilateral lower extremity injury	70 (66.0)
External fixation	56 (52.8)

*MVC* motor vehicle collision, *MCC* motorcycle collision, *FFS* fall from standing, *FFH* fall from height, *GSW* gunshot wound, *Peds versus auto* pedestrian versus automobile

Six-month patient-reported outcomes stratified by sex, age group, fracture pattern, and external fixation are summarized in Table 3. There were no significant differences in PROMIS scores, percent of normal, or BRS between males and females. Similarly, most survey outcomes did not differ significantly by age group (< 50 vs. ≥ 50 years), although younger patients reported significantly higher PI (62.7 vs. 59.3, *p* = 0.017) and lower GPH (38.8 vs. 42.4, *p* = 0.023) (Table 3). Stratification by fracture pattern (unicondylar vs. bicondylar) and external fixation status revealed no statistically significant differences; however, patients treated with external fixation trended toward lower PF scores (35.7 vs. 38.1, *p* = 0.079) and higher percent of normal function (56.7 vs. 48.0, *p* = 0.076) (Table 3).

**Table 3:** Six-month patient-reported outcome scores stratified by sex, age, fracture pattern, and external fixation status in patients with tibial plateau fractures

Domain	Male	Female	*p* value	Age < 50	Age ≥ 50	*p* value	Unicondylar	Bicondylar	*p* value	No ex-fix	ex-fix	*p* value
PF	37.8 (7.6)	35.8 (6.2)	0.141	36.2 (6.8)	37.4 (7.2)	0.384	37.9 (7.6)	36.3 (6.7)	0.278	38.1 (6.8)	35.7 (7.1)	0.079
PI	60.9 (6.9)	61.0 (7.6)	0.929	**62.7 (7.0)**	**59.3 (7.0)**	**0.017**	60.1 (7.5)	61.4 (7.1)	0.395	59.8 (7.4)	61.9 (7.0)	0.146
GPH	40.8 (8.3)	40.6 (8.2)	0.913	**38.8 (7.7)**	**42.4 (8.3)**	**0.023**	40.0 (7.5)	41.1 (8.6)	0.526	40.6 (8.1)	40.7 (8.4)	0.990
GMH	43.9 (8.0)	46.0 (8.7)	0.203	43.5 (7.5)	46.2 (9.0)	0.091	44.8 (8.7)	45.0 (8.3)	0.894	45.1 (8.9)	44.6 (8.0)	0.765
Depression	54.1 (11.9)	54.8 (10.4)	0.760	56.3 (11.3)	52.8 (10.8)	0.114	54.3 (11.0)	54.6 (11.3)	0.895	54.2 (11.3)	54.6 (11.1)	0.839
Anxiety	54.3 (11.4)	56.7 (10.4)	0.290	57.0 (11.5)	54.2 (10.3)	0.224	56.8 (10.0)	54.8 (11.4)	0.381	56.8 (10.4)	54.1 (11.3)	0.234
Percent of normal	53.6 (22.3)	51.4 (26.9)	0.667	52.6 (24.5)	52.6 (24.6)	0.994	49.9 (21.7)	53.9 (25.7)	0.448	48.0 (24.6)	56.7 (23.7)	0.076
BRS	3.5 (0.8)	3.5 (0.7)	0.840	3.5 (0.7)	3.4 (0.9)	0.704	3.55 (0.84)	3.46 (0.75)	0.591	3.43 (0.9)	3.54 (0.7)	0.494

*PF* physical function, *PI* pain interference, *GPH* global physical health, *GMH* global mental health, *BRS* brief resilience scale, *ex-fix* external fixation, *Age* < *50* less than 50 years of age, ≥ *50* greater than or equal to 50 years of age

Bolded values indicate statistical significance (*p* < 0.05)

When stratified by both sex and age, younger females reported higher PI (64.0 vs. 58.5, *p* = 0.009), lower GPH (37.5 vs. 43.1, *p* = 0.011), and lower anxiety (60.2 vs. 64.1, *p* = 0.046) than females ≥ 50 (Table 4). No statistically significant differences were found in PROMIS scores, percent of normal, or BRS between younger and older males (Table 4).

**Table 4:** Six-month patient-reported outcome scores by age group and sex in tibial plateau fracture patients

Domain	Female			Male		
	< 50 years, mean (SD)	≥ 50 years, mean (SD)	*p* value	< 50 years, mean (SD)	≥ 50 years, mean (SD)	*p* value
PF	35.5 (4.8)	36.0 (7.2)	0.781	36.8 (8.2)	38.8 (7.1)	0.327
PI	**64.0 (5.9)**	**58.5 (8.1)**	**0.009**	61.6 (7.9)	60.2 (5.8)	0.471
GPH	**37.5 (6.1)**	**43.1 (8.9)**	**0.011**	39.9 (8.8)	41.7 (7.9)	0.420
GMH	43.6 (6.7)	47.9 (9.8)	0.069	43.1 (8.2)	44.5 (8.0)	0.587
Depression	57.7 (8.1)	52.3 (11.6)	0.071	55.1 (13.5)	53.1 (10.1)	0.561
Anxiety	**60.2 (9.3)**	**64.1 (10.6)**	**0.046**	54.3 (12.6)	54.4 (10.3)	0.972
Percent of normal	53.9 (26.1)	49.1 (27.9)	0.557	51.5 (23.4)	55.6 (21.5)	0.506
BRS	3.68 (0.7)	3.35 (0.8)	0.125	3.38 (0.7)	3.56 (1.0)	0.441

*PF* physical function, *PI* pain interference, *GPH* global physical health, *GMH* global mental health, *BRS* brief resilience scale

Table 5 presents 6-month outcomes by Schatzker classification. Patients with Schatzker IV fractures reported significantly higher PI (67.7) compared to those with Schatzker I (63.1), II (57.4), III (65.5), and VI (61.1) fractures (*p* = 0.015) (Table 5). No significant differences were seen in other PROMIS scores, BRS, or percent of normal (Table 5).

**Table 5:** Six-month patient-reported outcome scores by Schatzker classification in patients with tibial plateau fractures

Domain	I mean (SD)	II mean (SD)	III mean (SD)	IV mean (SD)	VI mean (SD)	*p* value
PF	34.9 (8.3)	39.5 (7.7)	37.0 (2.7)	34.1 (6.6)	36.3 (6.9)	0.299
PI	**63.1 (6.9)**	**57.4 (7.5)**	**65.5 (2.2)**	**67.7 (4.0)**	**61.1 (7.1)**	**0.015**
GPH	37.3 (6.4)	41.9 (7.5)	37.4 (5.8)	35.3 (6.7)	41.2 (8.7)	0.336
GMH	47.4 (4.5)	45.8 (9.1)	42.6 (7.2)	39.8 (8.7)	45.0 (8.5)	0.577
Depression	51.7 (16.4)	53.8 (10.4)	60.1(4.0)	58.6 (9.9)	54.2 (11.4)	0.720
Anxiety	57.2 (15.9)	56.5 (9.5)	59.7 (4.9)	60.5 (9.7)	54.3 (11.4)	0.620
Percent of normal	44.8 (29.9)	52.0 (22.5)	39.2 (18.1)	38.2 (27.6)	55.7 (24.6)	0.319
BRS	3.03 (1.1)	3.53 (0.8)	3.73 (0.4)	3.93 (0.7)	3.46 (0.7)	0.415

*PF* physical function, *PI* pain interference, *GPH* global physical health, *GMH* global mental health, *BRS* brief resilience scale

When stratified by injury mechanism (Table 6), patients with high-energy injuries demonstrated significantly worse unadjusted GPH (39.4 vs. 43.5, *p* = 0.016), GMH (43.5 vs. 48.0, *p* = 0.010), and higher depression scores (55.9 vs. 51.2, *p* = 0.049). These differences remained statistically significant after adjusting for age, sex, and ISS: adjusted GPH (*p* = 0.014), GMH (*p* = 0.011), depression (*p* = 0.009). Additionally, following adjustment, patients with high-energy injuries demonstrated significantly higher anxiety scores (56.6 vs. 53.3, *p* = 0.015), lower percent of normal function (52.4 vs. 55.0, *p* = 0.002), and lower BRS scores (3.48 vs. 3.60, *p* = 0.004) (Table 6).

**Table 6:** Six-month patient-reported outcome scores by mechanism of injury in patients with tibial plateau fractures

Domain	Low energy, Unadj. mean (SD)	High energy, Unadj. mean (SD)	Unadj. *p* value	Low energy, Adj. mean* (95% CI)	High energy, Adj. mean* (95% CI)	Adj. *p* value*
PF	37.9 (5.8)	36.39 (7.5)	0.308	36.8 (33.4–40.2)	36.4 (34.5–38.3)	0.850
PI	58.9 (6.7)	61.88 (7.3)	0.052	60.6 (57.1–64.2)	61.7 (59.7–63.7)	0.611
GPH	**43.5 (8.5)**	**39.4 (7.8)**	**0.016**	**42.5 (38.6–46.3)**	**40.0 (37.9–42.2)**	**0.014**
GMH	**48.0 (8.8)**	**43.5 (7.9)**	**0.010**	**46.4 (42.5–50.4)**	**44.2 (42.0–46.4)**	**0.011**
Depression	**51.2 (11.1)**	**55.9 (10.9)**	**0.049**	**52.8 (47.3–58.4)**	**55.5 (52.4–58.6)**	**0.009**
Anxiety	52.6 (11.1)	56.8 (10.6)	0.076	**53.3 (47.9–58.6)**	**56.6 (53.5–59.7)**	**0.015**
Percent of normal	50.3 (24.9)	53.5 (24.3)	0.559	**55.0 (42.5–67.6)**	**52.4 (45.8–59.0)**	**0.002**
BRS	3.42 (0.8)	3.52 (0.7)	0.567	**3.60 (3.2–4.0)**	**3.48 (3.3–3.7)**	**0.004**

*PF* physical function, *PI* pain interference, *GPH* global physical health, *GMH* global mental health, *BRS* brief resilience scale, *unadj.* unadjusted, *adj.* adjusted

*Adjusted values derived from multivariable linear regression models adjusting for age, sex, and ISS

Bolded values indicate statistical significance (*p* < 0.05)

## Discussion

This study evaluated 6-month patient-reported outcomes following surgical fixation of tibial plateau fractures using PROMIS domains, percent of normal, and BRS scores. Younger patients, particularly females under 50, reported worse global physical health and greater pain interference compared to older counterparts. High-energy mechanisms of injury were associated with significantly lower global physical and mental health, and higher depression, even after adjusting for age, sex, and injury severity. These findings suggest that both patient- and injury-specific factors may influence recovery following tibial plateau fractures.

In this cohort, patients under 50 who underwent surgical fixation of tibial plateau fractures demonstrated worse global physical health and higher pain interference scores when compared to patients aged 50 and older. This trend was especially pronounced in younger females, who demonstrated higher PI and lower GPH relative to females aged 50 and older. This contrasts from Keating, who observed poorer functional outcomes in patients over 60 following surgical fixation of tibial plateau fractures [24]. Other studies, however, found no statistically significant differences in outcomes when stratified by age [25–27]. These mixed findings suggest a need for further investigation into the relationship between age and patient-reported outcomes following tibial plateau fractures.

Although external fixation may be used as part of staged management for more severe injuries, use of external fixation in this cohort was not associated with statistically significant differences in 6-month PROMIS domains, percent of normal function, or BRS scores compared with no external fixation management in this cohort.

When examining fracture characteristics, no significant differences in PROMIS scores, BRS, or percent of normal were observed when stratified by unicondylar versus bicondylar injuries. However, patients with Schatzker IV fractures reported significantly higher PI compared to other Schatzker types. Despite Schatzker VI fractures representing the most severe bony injury pattern with bicondylar involvement and metaphyseal-diaphyseal dissociation, these patients did not report the poorest outcomes as might be expected, possibly owing to worse soft tissue or articular injuries associated with Schatzker IV fractures as they are often viewed as knee dislocation equivalents [25, 26, 28–30]. These findings contrast Rademakers et al., who found that surgically treated unicondylar fractures had significantly better functional outcomes compared to bicondylar fractures, with Schatzker type VI fractures having the poorest outcome [26]. Barei et al. also found that severity of tibial plateau injury was significantly related to functional outcomes [29].

To contextualize these findings, the Minimal Clinically Important Difference (MCID) for PROMIS Physical Function after operatively treated tibial plateau fractures, estimated at 3.93 points, was considered [7]. In this cohort, most between-group PF differences were below this threshold (e.g., sex: 2.0 points; age < 50 vs. ≥ 50: − 1.2; unicondylar versus bicondylar: 1.6; external fixation: − 2.4; high- vs. low-energy, adjusted: − 0.4), suggesting limited clinical separation across these groups in physical function at 6 months. Although the absolute difference in PROMIS PF between Schatzker II and IV fractures exceeded the MCID (5.4 points), overall differences in physical function across Schatzker classifications did not reach statistical significance. While MCID values are typically derived for within-patient change, and therefore may not translate perfectly to between-group cross-sectional comparisons, MCID offers a clinically relevant benchmark for interpreting whether observed differences are likely to matter to patients in early recovery [7, 8].

In this cohort, most between-group differences in PROMIS domains were below MCID thresholds at 6 months. Prior PROMIS literature examining chronic medical conditions has demonstrated larger deviation from baseline PROMIS scores than those observed across orthopaedic injury subgroups in the present study [31–34], providing context for the effect sizes observed in this study.

In this study, patients sustaining high-energy injuries demonstrated significantly poorer global physical and mental health scores and higher depression scores compared to those with low-energy mechanisms. These differences remained statistically significant after adjustment for age, sex, and ISS. Furthermore, adjusted analyses revealed that patients sustaining high-energy mechanisms of injury demonstrated higher anxiety scores, lower percent of normal function, and lower BRS scores. Prior studies have demonstrated that high-energy mechanisms are associated with worse functional outcomes and increased complication rates across different fracture types compared to that of low-energy mechanisms [25, 35–37]. Additionally, the high prevalence of mental health conditions, including depression, anxiety, and post-traumatic stress symptoms in orthopedic trauma patients has been previously reported [38–42]. Together, these findings suggest that recovery following tibial plateau fractures is highly complex and encompasses both physical and psychological components.

### Limitations

This study has limitations that should be considered when interpreting these findings, including those inherent to its retrospective design. This study was conducted at a single institution, which may limit generalizability to other settings with different patient populations, clinical protocols, or tibial plateau management practices. While statistically significant differences in PROMIS scores, percent of normal, and BRS were observed, the clinical relevance of these findings is less clear. This is due, in part, to the limited availability of established MCID thresholds for specific PROMIS domains, particularly within orthopaedic trauma populations. Furthermore, surveys were administered electronically, and only 6-month scores were evaluated in this study, which may not fully capture the longitudinal trajectory of functional recovery or outcomes. Because this analysis relied on complete survey data, patients who did not complete PROMIS assessments were excluded, which may limit the generalizability of our findings and could have influenced the observed results in this study.

## Conclusion

Patient-reported recovery following tibial plateau fractures varied across patient- and injury-specific characteristics. Younger patients, particularly females under 50, demonstrated greater pain interference and lower global physical health, while high-energy mechanisms of injury were independently associated with worse physical and psychological outcomes across multiple domains, even after adjustment for age, sex, and injury severity. These findings support an approach to postoperative care that addresses both functional recovery and mental well-being.

## Supplementary Information

Below is the link to the electronic supplementary material.

Supplementary Material 1

## Data Availability

No datasets were generated or analysed during the current study.

## References

[1] Donovan RL et al (2022) Epidemiology and outcomes of tibial plateau fractures in adults aged 60 and over treated in the United Kingdom. Injury 53(6):2219–2225 35367077 10.1016/j.injury.2022.03.048

[2] Reátiga Aguilar J et al (2022) Epidemiological characterization of tibial plateau fractures. J Orthop Surg Res 17(1):106 35183211 10.1186/s13018-022-02988-8 PMC8858541

[3] Rozell JC et al (2016) Tibial plateau fractures in elderly patients. Geriatr Orthop Surg Rehabil 7(3):126–134 27551570 10.1177/2151458516651310 PMC4976737

[4] Egol K, Koval KJ, Zuckerman J (2014) Handbook of fractures. Lippincott Williams & Wilkins, Philadelphia

[5] Gálvez-Sirvent E, Ibarzábal-Gil A, Rodríguez-Merchán EC (2022) Complications of the surgical treatment of fractures of the tibial plateau: prevalence, causes, and management. EFORT Open Rev 7(8):554–568 35924649 10.1530/EOR-22-0004 PMC9458943

[6] Taiwo BS et al (2025) At the peak: a review of current diagnostic and therapeutic concepts surrounding tibial plateau fractures. Orthop Trauma. 10.1016/j.mporth.2025.01.005

[7] Thorne TJ et al (2024) The MCID of the PROMIS physical function instrument for operatively treated tibial plateau fractures. Injury 55(4):111375 38290908 10.1016/j.injury.2024.111375 PMC11351672

[8] Hammert WC, Calfee RP (2020) Understanding promis. J Hand Surg 45(7):650–654 10.1016/j.jhsa.2020.03.016 32370909

[9] Ader DN (2007) Developing the patient-reported outcomes measurement information system (PROMIS). Med Care 45:S1–S2 17443116 10.1097/01.mlr.0000258615.42478.55 PMC2829758

[10] Riley WT et al (2010) Patient-reported outcomes measurement information system (PROMIS) domain names and definitions revisions: further evaluation of content validity in IRT-derived item banks. Qual Life Res 19:1311–1321 20593306 10.1007/s11136-010-9694-5 PMC3670674

[11] Brodke DJ, Saltzman CL, Brodke DS (2016) PROMIS for orthopaedic outcomes measurement. J Am Acad Orthop Surg 24(11):744–749 27661391 10.5435/JAAOS-D-15-00404

[12] Cavallero M et al (2018) Locking plate fixation in a series of bicondylar tibial plateau fractures raises treatment costs without clinical benefit. J Orthop Trauma 32(7):333–337 29738401 10.1097/BOT.0000000000001188

[13] Ochen Y et al (2020) Long-term outcomes after open reduction and internal fixation of bicondylar tibial plateau fractures. Injury 51(4):1097–1102 32147141 10.1016/j.injury.2020.03.003

[14] Sato EH et al (2022) Meniscus tear requiring intra-operative repair does not influence mid-term patient reported outcomes in operatively treated tibial plateau fractures. J Orthop Trauma 38:109–114 10.1097/BOT.0000000000002724 38031250

[15] Stenquist DS et al (2025) Fracture characteristics and functional outcomes for Schatzker V/VI bicondylar tibial plateau fractures with a separate tubercle fragment: a comparative study. Arch Orthop Trauma Surg 145(1):1–9 10.1007/s00402-024-05660-4 39774982

[16] Virkus WW et al (2018) Costs and complications of single-stage fixation versus 2-stage treatment of select bicondylar tibial plateau fractures. J Orthop Trauma 32(7):327–332 29920192 10.1097/BOT.0000000000001167

[17] O’Neill DC et al (2025) Patient-reported outcomes after tibial plateau fracture: infection confers greatest risk of poor outcome. Eur J Orthop Surg Traumatol 35(1):1–8 10.1007/s00590-024-04160-w 39666150

[18] Smith BW et al (2008) The brief resilience scale: assessing the ability to bounce back. Int J Behav Med 15(3):194–200 18696313 10.1080/10705500802222972

[19] Parry JA et al (2021) Percent of normal: a pragmatic patient-reported outcome measure for the orthopaedic trauma clinic. J Orthop Trauma 35(11):e429–e432 33591064 10.1097/BOT.0000000000002078

[20] Whiting PS et al (2015) What factors influence follow-up in orthopedic trauma surgery? Arch Orthop Trauma Surg 135(3):321–327 25617213 10.1007/s00402-015-2151-8

[21] Zelle BA et al (2013) Loss of follow-up in orthopaedic trauma: Is 80% follow-up still acceptable? J Orthop Trauma 27(3):177–181 23449099 10.1097/BOT.0b013e31825cf367

[22] Boyd CR, Tolson MA, Copes WS (1987) Evaluating trauma care: the TRISS method. J Trauma Acute Care Surg 27(4):370–378 3106646

[23] Butcher N, Balogh Z (2014) Update on the definition of polytrauma. Eur J Trauma Emerg Surg 40:107–111 26815890 10.1007/s00068-014-0391-x

[24] Keating JF (1999) Tibial plateau fractures in the older patient. Bulletin (Hosp Jt Dis NY) 58(1):19–23 10431630

[25] Hap DXF, Kwek EBK (2020) Functional outcomes after surgical treatment of tibial plateau fractures. J Clin Orthop Trauma 11:S11–S15 31992910 10.1016/j.jcot.2019.04.007 PMC6977533

[26] Rademakers M et al (2007) Operative treatment of 109 tibial plateau fractures: five-to 27-year follow-up results. J Orthop Trauma 21(1):5–10 17211262 10.1097/BOT.0b013e31802c5b51

[27] Kim J-K et al (2021) Comparison of tibial plateau fracture surgical outcomes between young and elderly patients: are outcomes really poorer in the elderly? Arch Orthop Trauma Surg. 10.1007/s00402-021-03855-7 33689018 10.1007/s00402-021-03855-7

[28] Manidakis N et al (2010) Tibial plateau fractures: functional outcome and incidence of osteoarthritis in 125 cases. Int Orthop 34(4):565–570 19440710 10.1007/s00264-009-0790-5 PMC2903147

[29] Barei DP et al (2006) Functional outcomes of severe bicondylar tibial plateau fractures treated with dual incisions and medial and lateral plates. J Bone Joint Surg 88(8):1713–1721 16882892 10.2106/JBJS.E.00907

[30] Jagdev SS et al (2018) Functional outcome and incidence of osteoarthritis in operated tibial plateau fractures. Arch Bone Jt Surg 6(6):508 30637306 PMC6310186

[31] Rothrock NE et al (2010) Relative to the general US population, chronic diseases are associated with poorer health-related quality of life as measured by the patient-reported outcomes measurement information system (PROMIS). J Clin Epidemiol 63(11):1195–1204 20688471 10.1016/j.jclinepi.2010.04.012 PMC2943571

[32] Pak SS, Miller MJ, Cheuy VA (2021) Use of the PROMIS-10 global health in patients with chronic low back pain in outpatient physical therapy: a retrospective cohort study. J Patient Rep Outcomes 5(1):81 34487270 10.1186/s41687-021-00360-8 PMC8421489

[33] Homco J et al (2019) Variation and change over time in PROMIS-29 survey results among primary care patients with type 2 diabetes. J Patient Cent Res Rev 6(2):135–147 31414025 10.17294/2330-0698.1694 PMC6676762

[34] Schnall R et al (2017) A health-related quality-of-life measure for use in patients with HIV: a validation study. AIDS Patient Care STDS 31(2):43–48 28051875 10.1089/apc.2016.0252 PMC5312551

[35] van der Vliet QMJ et al (2019) Polytrauma and high-energy injury mechanisms are associated with worse patient-reported outcomes after distal radius fractures. Clin Orthop Relat Res 477(10):2267–2275 30985610 10.1097/CORR.0000000000000757 PMC6999931

[36] Cecil A et al (2020) High- versus low-energy acetabular fracture outcomes in the geriatric population. Geriatr Orthop Surg Rehabil 11:2151459320939546 32733771 10.1177/2151459320939546 PMC7370335

[37] Dyskin E et al (2019) A survey of high- and low-energy acetabular fractures in elderly patients. Geriatr Orthop Surg Rehabil 10:2151459319870426 31456902 10.1177/2151459319870426 PMC6702768

[38] Houwen T et al (2022) There are more things in physical function and pain: a systematic review on physical, mental and social health within the orthopedic fracture population using PROMIS. J Patient Rep Outcomes 6(1):34 35384568 10.1186/s41687-022-00440-3 PMC8986932

[39] Scott S et al (2024) Prevalence, resources, provider insights, and outcomes: a review of patient mental health in orthopaedic trauma. J Orthop Surg Res 19(1):538 39223649 10.1186/s13018-024-04932-4 PMC11370264

[40] Bhandari M et al (2008) Psychological distress and quality of life after orthopedic trauma: an observational study. Can J Surg 51(1):15 18248701 PMC2386305

[41] Jella TK, Cwalina TB, Vallier HA (2022) Concurrent mental illness and financial barriers to mental health care among a nationally representative sample of orthopaedic trauma survivors. J Orthop Trauma 36(12):665–673 36399680 10.1097/BOT.0000000000002433

[42] Crichlow RJ et al (2006) Depression in orthopaedic trauma patients: prevalence and severity. J Bone Jt Surg 88(9):1927–1933 10.2106/JBJS.D.02604 16951107

